# Use of field‐portable ultrasonography reveals differences in developmental phenology and maternal egg provisioning in two sympatric viviparous snakes

**DOI:** 10.1002/ece3.3928

**Published:** 2018-02-19

**Authors:** Amanda M. Sparkman, Kenneth R. Chism, Anne M. Bronikowski, Lilly J. Brummett, Lucia L. Combrink, Courtney L. Davis, Kaitlyn G. Holden, Nicole M. Kabey, David A. W. Miller

**Affiliations:** ^1^ Department of Biology Westmont College Santa Barbara CA USA; ^2^ Department of Ecology, Evolution and Organismal Biology Iowa State University Ames IA USA; ^3^ Department of Ecosystem Science and Management Pennsylvania State University University Park PA USA; ^4^ Intercollege Graduate Ecology Program Pennsylvania State University University Park PA USA

**Keywords:** embryonic development, reproduction, *Thamnophis*, ultrasonography, viviparity

## Abstract

A thorough understanding of the life cycles underlying the demography of wild species is limited by the difficulty of observing hidden life‐history traits, such as embryonic development. Major aspects of embryonic development, such as the rate and timing of development, and maternal–fetal interactions can be critical features of early‐life fitness and may impact population trends via effects on individual survival. While information on development in wild snakes and lizards is particularly limited, the repeated evolution of viviparity and diversity of reproductive mode in this clade make it a valuable subject of study. We used field‐portable ultrasonography to investigate embryonic development in two sympatric garter snake species, *Thamnophis sirtalis* and *Thamnophis elegans* in the Sierra Nevada mountains of California. This approach allowed us to examine previously hidden reproductive traits including the timing and annual variation in development and differences in parental investment in young. Both species are viviparous, occupy similar ecological niches, and experience the same annual environmental conditions. We found that *T. sirtalis* embryos were more developmentally advanced than *T. elegans* embryos during June of three consecutive years. We also found that eggs increased in volume more substantially across developmental stages in *T. elegans* than in *T. sirtalis*, indicating differences in maternal provisioning of embryos via placental transfer of water. These findings shed light on interspecific differences in parental investment and timing of development within the same environmental context and demonstrate the value of field ultrasonography for pursuing questions relating to the evolution of reproductive modes, and the ecology of development.

## 1 INTRODUCTION

The ability to accurately assess population‐level outcomes of demographic changes requires measuring responses across the full life cycle of demographic events that make up a species’ life history. This applies both to evolutionary‐ecological applications (e.g., understanding how life‐history differences evolve; Cole, 1954) and measuring fitness in wild populations (McGraw & Caswell, 1996) as well as conservation applications, such as identifying sources and sinks in spatially structured populations (Runge, Runge, & Nichols, 2006) and assessing limiting factors across an animal's life‐cycle (Marra, Cohen, Loss, Rutter, & Tonra, 2015). In many cases, observing complete life cycles of plants and animals in the wild can be difficult if not impossible. Examples of hidden or unobservable demographic variables that have plagued researchers’ ability to make strong inferences include partitioning mortality among seasons in migratory species (Marra et al., 2015), quantifying and following long‐distance dispersal events (Cain, Milligan, & Strand, 2000), and observing life‐history stages that occur underground (Muñoz, Miller, Sutherland, & Grant, 2016). The ability of researchers to fill these information gaps come from some combination of creative, technical, and methodological innovation (e.g., Carlo, García, Martínez, Gleditsch, & Morales, 2013; Hobson, 1999; Muñoz et al., 2016; Stutchbury et al., 2009).

Embryonic development is a crucial and usually unobserved life‐history stage in many wild species. The environmental conditions under which embryonic development occurs can have profound impacts on early‐life fitness (Lindström, 1999; Monaghan, 2008). Temperature and water availability during development, as well as timing of birth or hatching, for instance, can affect neonatal sex, growth rate, performance, and survival (e.g., Brooks, Bobyn, Galbraith, Layfield, & Nancekivell, 1991; Janzen, 1995; Lourdais, Shine, Bonnet, Guillon, & Naulleau, 2004; Olson, Vleck, & Vleck, 2006; Uller & Olssen, 2010; Lorioux et al., 2013). At the same time, genetic variation in timing and rate of development can occur within and across species, such that developmental phenotypes are determined by complex interactions between genetic and environmental factors (reviewed in Sultan, 2007). Recently, there has been increasing interest in the dynamics of development in the wild, particularly with rising concern regarding effects of climate change on reproductive phenology (Abouheif et al., 2014; Gilbert, Bosch, & Ledón‐Rettig, 2015; Ledón‐Rettig & Pfennig, 2011; Parmesan, 2007; Sultan, 2007; Telemeco, Elphick, & Shine, 2009; Walther et al., 2002; Winkler, Dunn, & McCulloch, 2002). Shifts in the onset of breedings seasons, length of developmental periods, and hatching times have been documented in a wide range of species, including insects, amphibians, turtles, lizards, and birds (e.g., Edge et al., 2017; Parmesan, 2007; Rutschmann et al., 2016; Urban, Richardson, & Freidenfelds, 2014; Valtonen, Latja, Leinonen, & Pöysä, 2017). Yet much remains to be learned regarding plasticity and phylogenetic constraints on developmental life history in natural populations with varying reproductive modes, including viviparity (live‐bearing) and oviparity (egg‐bearing) species (Sultan, 2007).

One of the major challenges in studying development in the wild is that while gravidity or pregnancy can be fairly straightforward to diagnose in many cases, it is difficult to noninvasively obtain more fine‐grained information about embryonic development in the field. Often the timing of fertilization, duration of gestation, and location of oviposition/birth are unknown, particularly for more secretive species. Furthermore, while impressive progress has been made studying the dynamics of development in the laboratory in a range of species, many basic features, such as plastic differences in developmental rate and environmentally‐mediated variation in maternal–fetal interactions, have remained largely unstudied in wild populations. Ultrasonography has been used to great effect in monitoring development in humans, laboratory, domestic and zoo animals (Hildebrandt, Brown, Hermes, & Goritz, 2003; Hildebrandt, Göritz, & Hermes, 2006), but use in the field has been more limited. However, field‐portable ultrasound systems can now offer high‐resolution images of development in a range of species, large and small, and are well‐posed to help fill in gaps in our knowledge on development in the wild and provide valuable information for demographic and fitness analyses (Gilman & Wolf, 2007; Hildebrandt, Hermes, Jewgenow, & Göritz, 2000).

Squamate reptiles—snakes and lizards—are a particularly important and interesting group to improve our understanding of development in the wild. First, there is remarkable diversity of reproductive mode within this group, as viviparity has independently evolved from oviparity more than 100 times (Shine, 1983; Blackburn, 2006). Furthermore, while even viviparous snakes and lizards remain primarily lecithotrophic (i.e., nutrition is derived primarily from yolk), there is extensive variation in the degree of maternal provisioning via placenta (Blackburn, 2006). Second, while some important work has been done linking environmental factors to lizard developmental phenology in the wild, only very limited data are available for snakes (Urban et al., 2014). As snakes and lizards are ectotherms and may be particularly vulnerable to changes in environmental temperatures, an understanding of how a changing environment affects development in these species is a critical component of understanding demographic and fitness consequences of climate change. Finally, ultrasonography has already been shown to be a highly effective tool in reptiles, providing valuable information on clutch/litter size, egg mass, and developmental stage (e.g., Gilman & Wolf, 2007; Stahlschmidt, Brashears, & Denardo, 2011; Lorioux et al., 2013; Lourdais et al., 2015).

While many studies have focused on energy allocation to eggs, the dynamics of water flux into eggs over the course of development has become increasingly of greater interest (e.g., Packard, Tracy, & Roth, 1977; Deeming, 2006; Brown & Shine, 2009; Dupoué et al., 2015; Lourdais et al., 2015; Bonnet, Naulleau, & Shine, 2017). Water is required for the conversion of yolk to embryonic tissue in both oviparous and viviparous species (Thompson & Speake, 2002; Vleck, 1991), and water balance has critical ramifications for embryonic survival (e.g., Aubret, Bonnet, Shine, & Maumelat, 2003; Cagle, Packard, Miller, & Packard, 1993; Vleck, 1991; Warner & Andrews, 2002). Recent work in the viviparous aspic viper (*Vipera aspis*) has made ground‐breaking use of ultrasonography to monitor changes in egg volume over gestation in captivity. They found that eggs can increase in size by over 200%, primarily due to maternal water transfer (Lourdais et al., 2015). Other research on *V. aspis* has also shown that there is a trade‐off between maternal and fetal hydration, such that when mothers are water‐deprived, they become dehydrated, but their eggs derive water via placental transfer to the same degree as eggs in nondehydrated mothers (Dupoué et al., 2015). In general, these findings call for further study of maternal water provisioning in other viviparous species, so that we can begin to understand how fluctuations in water availability might affect developing embryos in the wild.

**Figure 1:**
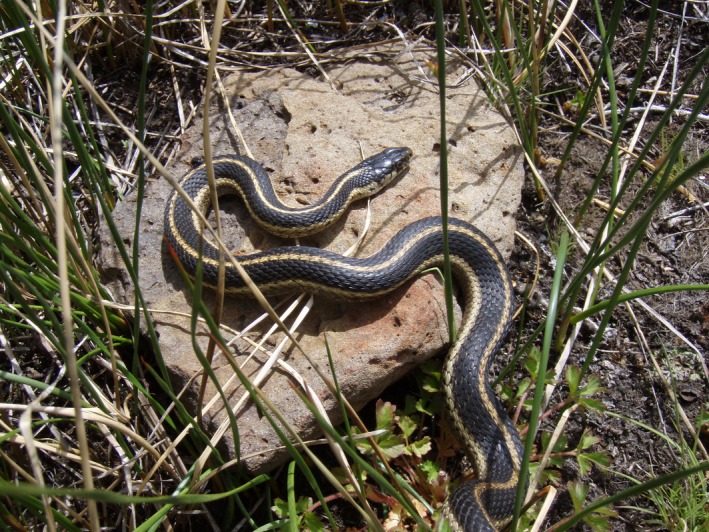
Gravid female western terrestrial garter snake (*Thamnophis elegans*). Photograph taken by A.M. Sparkman

We use two species of garter snake inhabiting discrete montane meadow habitats to begin an investigation of the relative effects of environmental and phylogenetic factors on embryonic development in the wild. These two species, *Thamnophis sirtalis* (common garter snake) and *T. elegans* (western terrestrial garter snake) (Figure 1), are estimated to have diverged around 6.9 million years ago; *T. sirtalis* is part of a small clade that is an outgroup to all other garter snakes species, including *T. elegans* (Manier & Arnold, 2005; de Queiroz, Lawson, & Lemos‐Espinal, 2002). In the Sierra Nevada mountains in Lassen County, California, these two species share hibernation and retreat sites, bask in the same grasses throughout the day, and show an almost complete overlap in diet within meadows (Kephart, 1982). Furthermore, these sympatric species experience the same annual fluctuations in photoperiod, temperature, and precipitation, as well as prey availability. Both species are capital breeders, with frequency of reproduction highly contingent on availability of anuran prey the previous year (Bronikowski & Arnold, 1999; Miller, Clark, Arnold, & Bronikowski, 2011). As such, this system forms a kind of natural experiment in which to explore development in two closely related species in the context of the same general environmental conditions.

Both *T. sirtalis* and *T. elegans*, like all garter snakes, are viviparous. While garter snake embryos are primarily lecithotrophic, with the yolk being the major source of nutrients, functional studies suggest that there is placental transfer of respiratory gases, nutrients, and water from the mother to the developing egg (Blackburn & Lorenz, 2003a,b; Blackburn, Stewart, Baxter, & Hoffman, 2002; Hoffman, 1970; Stewart, Blackburn, Baxter, & Hoffman, 1990). Furthermore, morphological studies of the placenta in *T. sirtalis, T. ordinoides,* and *T. radix* (the latter both closely related to *T. elegans*—de Queiroz et al., 2002) indicate that maternal and fetal components of the placenta become more vascularized during the last half of gestation; furthermore, the shell membrane (used for shell deposition in oviparous species, but still present in viviparous species) shows a reduction in thickness as gestation progresses, bringing maternal and fetal tissues into closer proximity (Blackburn & Lorenz 2003b; Blackburn & Flemming, 2009;). Thus, prior evidence points to the likelihood that garter snakes, like many other viviparous reptiles, will show evidence of maternal provisioning during development.

Our study employed field‐portable ultrasonography to test whether sympatric *T. elegans* and *T. sirtalis* showed similar developmental phenology and evidence of maternal provisioning of embryos. We compared distributions of females with embryos at different developmental stages between species over a given time period, and tested for differences in egg volume across developmental stages. We hypothesized that similarities in both phenology and maternal provisioning could result from living under the same general environmental conditions. Alternatively, differences in phenology and provisioning could indicate interspecific differences in genes or gene by environment interactions underlying developmental phenotypes. In pursuing these questions, we provide not only basic information regarding dynamics of development in this system, but also demonstrate the feasibility and value of a method for determining otherwise hidden patterns of developmental life history in the wild within and across species and environments.

## 2 METHODS

### 2.1 Study system and ultrasonography

All procedures in this study were conducted with approval from the Iowa State Institutional Animal Care and Use Committee and a California Department of Fish and Wildlife Scientific Collecting Permit. Pregnant *T. sirtalis* and *T. elegans* females were captured by hand from five distinct montane meadows (PAP, NAM, MAH, SUM, and PVM from Manier et al., 2005) in the vicinity of Eagle Lake in Lassen County in the Sierra Nevada Mountains of California. This study was conducted from 2015 to 2017 during a roughly one‐month interval from early June to early July in each year.

**Figure 2:**
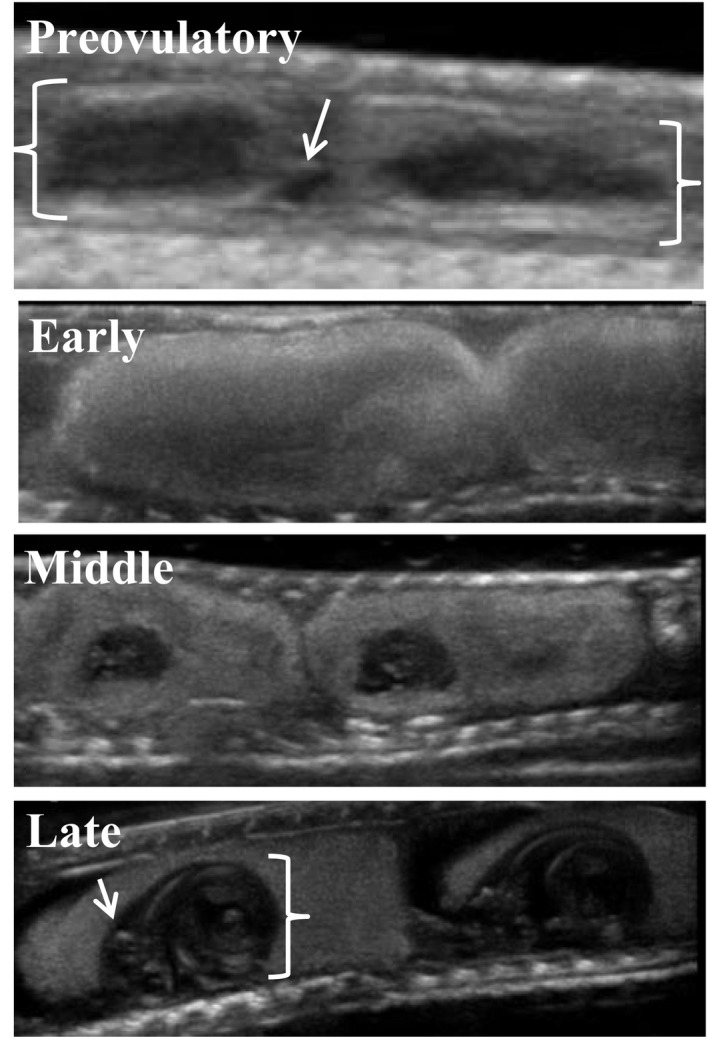
Ultrasound images of four general developmental stages for follicles/eggs of viviparous female garter snakes. The image of preovulatory follicles shows an anechoic (dark appearing) previtelligenic follicle (arrow) flanked by two follicles of different size in the process of vitellogenesis (brackets). After ovulation, early development eggs show no discernable embryos, whereas middle and late development show clear, echogenic embryonic skeletal elements within the embryonic sac, with late development embryos showing a clear cranium (arrow) and vertebral coil (bracket)

On the day of or following capture, females were scanned with a field‐portable Sonosite M‐Turbo Ultrasound (Fujifulm SonoSite, Inc.) with a 15 MHz transducer. During the ultrasound, each female was gently restrained with her abdomen submerged in a water bath. Eggs/follicles, which are positioned linearly caudal to the gall bladder in garter snakes, were counted and classified into the following general developmental stages: preovulatory, early development, middle development, and late development (Figure 2). Vitellogenesis is the process of yolk deposition into the oocyte prior to ovulation from the ovary. Preovulatory vitellogenic follicles were identified as less echogenic (i.e., bright‐appearing) than ovulated eggs, more variable in size, irregularly shaped, and often overlapping and interspersed with anechoic (i.e., dark‐appearing) prevoulatory follicles (Gilman & Wolf, 2007; Matayoshi, de Souza, Ferreira, Prestes, & dos Santos, 2012; Prades, Lastica, & Acorda, 2013; Schumacher & Toal, 2001). Early, middle, and late developmental stages corresponded roughly to Zehr's 1–10, 11–24, and 25–32 developmental stages for *Thamnophis sirtalis*, respectively (Zehr, 1962), and represent broader groupings of Zehr's more fine‐grained categories. Postovulatory eggs with no embryo visible were classified as early development. Eggs classified as middle development had an embryonic sac clearly visible (though still small relative to the yolk sac), with indistinct echogenic skeletal elements. Eggs classified as late development had prominent, enlarged embryonic sacs with the cranium and vertebral coil evident. Eggs within females appeared to be in the same stage of development, allowing us to classify all embryos for a given female as a single developmental stage (although note that in some reptiles, differences in developmental stage can be evident among embryos within the same female—see Gilman & Wolf, 2007).

**Figure 3:**
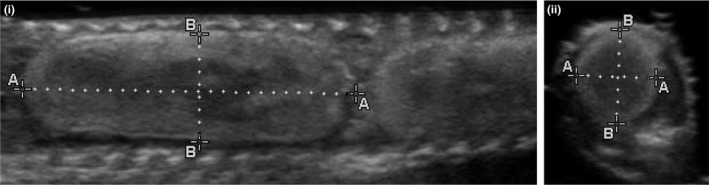
Ultrasound images depicting garter snake egg measurements made with virtual calipers. Lateral scans (i) were used to measure (i‐A) egg length and (i‐B) width. Transverse scans were used to measure (ii‐A) egg height and (ii‐B) egg width, which can also be measured with a transverse scan

Egg volume (*V*) was estimated by conducting both lateral and ventral scans to measure egg length (*l*; cranial‐caudal), width (*w*; left‐right), and height (*h*; dorsal‐ventral). Measurements were made using virtual calipers on still images. To calculate volume, measurements were applied to the volumetric equation for a scalene ellipsoid, V=(3/4)πlwh (Stahlschmidt et al., 2011; Figure 3). We gently rotated the transducer to different angles to obtain images where maximal measurements for each egg dimension could be observed. Egg volume was calculated for the first and last eggs of each female, and averaged to obtain a mean egg volume.

While this study was cross‐sectional in design, we recaptured and subsequently ultrasounded two *T. elegans* females and one *T. sirtalis* females again at later dates within the same year. We provide information on changes in developmental stage and egg volume within these individuals, for comparison with our findings based on the cross‐sectional analysis.

### 2.2 Statistical analyses

#### 2.2.1 Developmental phenology

All statistical analyses were performed using SAS 9.3 (SAS Institute Inc). We tested for differences in developmental stage between the two species using logistic regression (PROC LOGISTIC), as we had a robust sample size of independent observations and independent variables that were not correlated with each other. The full sample size included 255 females. Sample sizes were low for 2015 (*T. sirtalis N* = 6; *T. elegans N* = 17), but as the two species exhibited the same trends in 2015 as in subsequent years, and the analysis remained consistent whether or not they were included, we retained them in our analysis. Sample sizes were substantially higher for both 2016 (*T. sirtalis N* = 18; *T. elegans N* = 63) and 2017 (*T. sirtalis N* = 54; *T. elegans N* = 99). The response variable, developmental stage, had four levels (preovulatory, early development, middle development, and late development). We tested for main effects of species, year, and source population. We also included ultrasound date in Julian days as a covariate, to control for potential temporal differences in capture rate between species over the study period, as well as interactions with species and each of the other three variables.

#### 2.2.2 Maternal provisioning

We tested for evidence of maternal provisioning by testing for differences in egg volume among postovulatory developmental stages in both species, using 208 females derived from all six populations in 2016 (*T. sirtalis N* = 17; *T. elegans N* = 58) and 2017 (*T. sirtalis N* = 53; *T. elegans N* = 80). Note that 2015 was not included for egg volume analysis, as not all measurements required to calculate volume were made in this year. We conducted an ANCOVA analysis using PROC GLM with mean egg volume as the response variable, and species, developmental stage (early, middle, late), source population, and year as main effects. Egg volume showed normal distribution and observations were independent, satisfying the major assumptions of ANCOVA. Given that maternal body size can influence egg size and a trade‐off can exist between the number and size of offspring (Seigel & Ford, 1987), we also included maternal snout‐vent length (SVL) and litter size (i.e., number of eggs counted during the ultrasound) as covariates. We tested for two‐way interactions between species and all other main effects.

## 3 RESULTS

### 3.1 Developmental phenology

**Figure 4:**
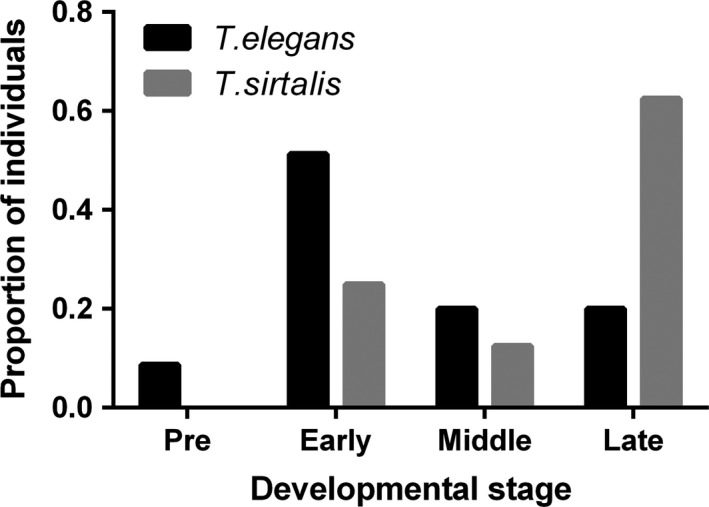
Proportion of females with follicles/eggs in preovulatory (pre), early development, middle development, and late development stages for both *Thamnophis elegans* and *Thamnophis sirtalis* pooled across years

Species, source population, and ultrasound date were all highly significantly associated with developmental stage (Table 1). *T. sirtalis* embryos were at more advanced developmental stages over the study period than *T. elegans* embryos, with the majority of *T. sirtalis* females having embryos at middle or late stages of development, but the majority of *T. elegans* females having embryos at the early stage of development (Figure 4). Populations also varied in proportion of individuals in different developmental stages. Furthermore, more advanced developmental stages were more prevalent at later dates. We dropped all interactions between species and other variables, as well as year from the final model, as these were nonsignificant. It is worth noting, however, that there were 13 *T. elegans* individuals in 2017 that had preovulatory vitellogenic follicles, a stage not observed in any other year.

**Table 1:** Results from logistic regression analysis of developmental stage in *Thamnophis elegans* and *Thamnophis sirtalis,* detailing the degrees of freedom (*df*), chi‐square statistic (χ^2^), and *p*‐value (*p*)

Effect	*df*	χ^2^	*p*
Species	3	79.01	<.0001
Source population	12	37.60	<.0001
Ultrasound day	3	71.86	<.0001

Note that for some populations, one or the other species was not sampled each year. As we detected a source population difference in developmental stages, we also ran a similar analysis restricted only to the two populations, MAH and PAP, from which we consistently had representatives of both species, to confirm that our species differences could not simply be explained by population differences. Our findings remained the same, with differences in distribution of developmental stages for each species being very similar in the restricted compared to the full analysis; thus, we present only the full analysis including all populations here.

### 3.2 Maternal provisioning

**Figure 5:**
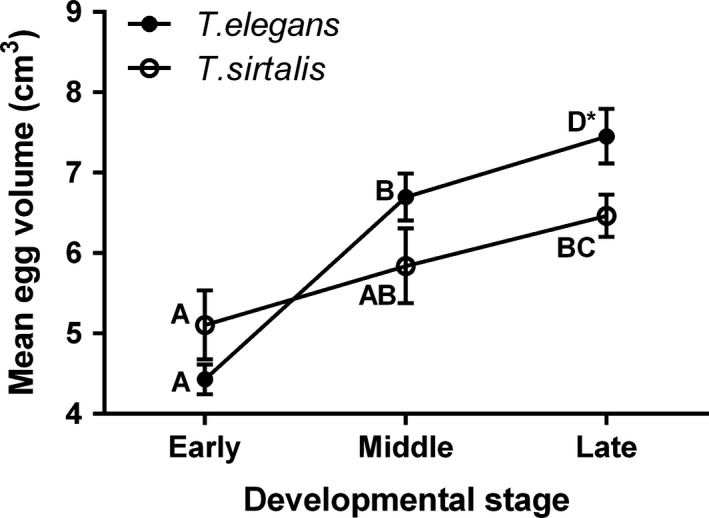
Least square means and standard errors of egg volume across developmental stages for *Thamnophis sirtalis* and *Thamnophis elegans* females. Different letters denote means that are significantly different. The asterisk (*) by “D” denotes the fact that late development *T. elegans* eggs were only marginally significantly different from middle development *T. elegans* eggs

The final model for mean egg volume included species, developmental stage, source population, body size, litter size, and the interactions between species and developmental stage, and species and body size (Table 2). Year and species x litter size were nonsignificant and thus excluded from the final model (although note that results from other variables remained consistent when included). The significant species x developmental stage interaction revealed that *T. sirtalis* and *T. elegans* differed in the manner in which volume differed among eggs in different developmental stages. Comparisons of least square means revealed that there was a significant 51% increase in volume, on average, in *T. elegans* eggs between early and middle development, and a marginally significant 11% increase (*p = *.06) between middle and late development eggs (Figure 5). In contrast, a more modest 14% increase in volume on average occurred between early and middle development for *T. sirtalis* eggs, with an additional 11% increase between middle and late development. Overall, *T. elegans* eggs showed a 68% increase in volume between early and late developmental stages, whereas *T. sirtalis* showed a 27% increase. Thus, while egg volume at the early developmental stage did not differ between species, *T. elegans* eggs were significantly larger than *T. sirtalis* eggs by late development.

**Table 2:** Results from ANOVA of mean egg volume of *Thamnophis sirtalis* and *Thamnophis elegans* as estimated by ultrasonography, detailing each main effect and respective degrees of freedom (*df*), *F*‐statistic (*F*), and *p*‐value (*p*)

Effect	*df*	*F*	*p*
Species	1,195	4.44	.036
Source population	4,195	3.60	.007
Body size	1,195	26.12	<.0001
Developmental stage	2,195	28.82	<.0001
Litter size	1,195	9.32	.003
Species × body size	1,195	3.96	.048
Species × developmental stage	2,195	5.05	.007

**Figure 6:**
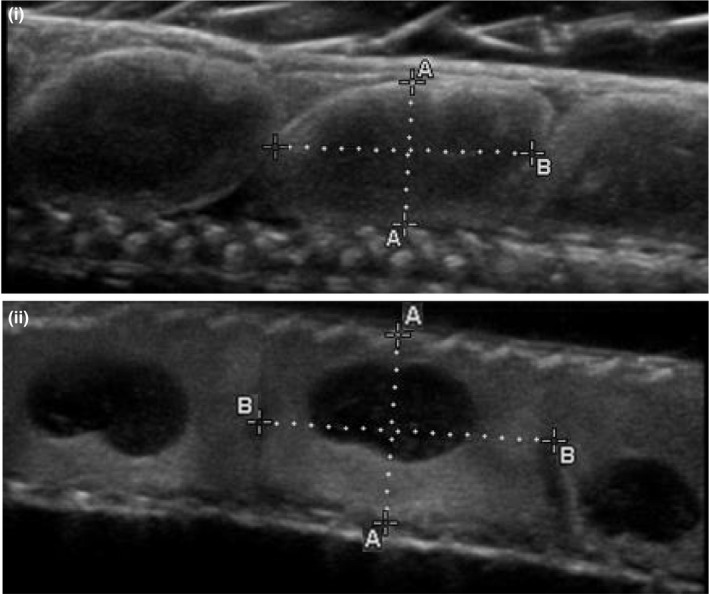
Ultrasound image of eggs within the same *Thamnophis elegans* female captured on (i) 16 June 2016, and (ii) again on 7 July 2016. Embryonic development in (ii) is more advanced than that shown in (i), and this image highlights the (approx. 92%) increase in volume, with eggs more tightly packed against the confines of the maternal abdomen and adjacent eggs

The three recaptured females showed species‐specific patterns of change in egg volume over time that were generally consonant with our cross‐sectional data—although of course this is a low sample size and therefore should be considered primarily as anecdotal support of our primary analysis. One of the *T. elegans* female with eggs at the early development stage during both captures showed an increase in egg volume of 27% over 7 days. In contrast, eggs in the recaptured *T. sirtalis* female showed only a 2% increase in egg volume over 8 days, while progressing from early to middle development. The second *T. elegans* female showed a 92% increase in egg volume over 21 days, during which the embryos progressed from early to middle development. The change in volume and developmental stage was immediately evident via ultrasound, with eggs appearing more tightly packed in the maternal abdomen at the later date (Figure 6).

**Figure 7:**
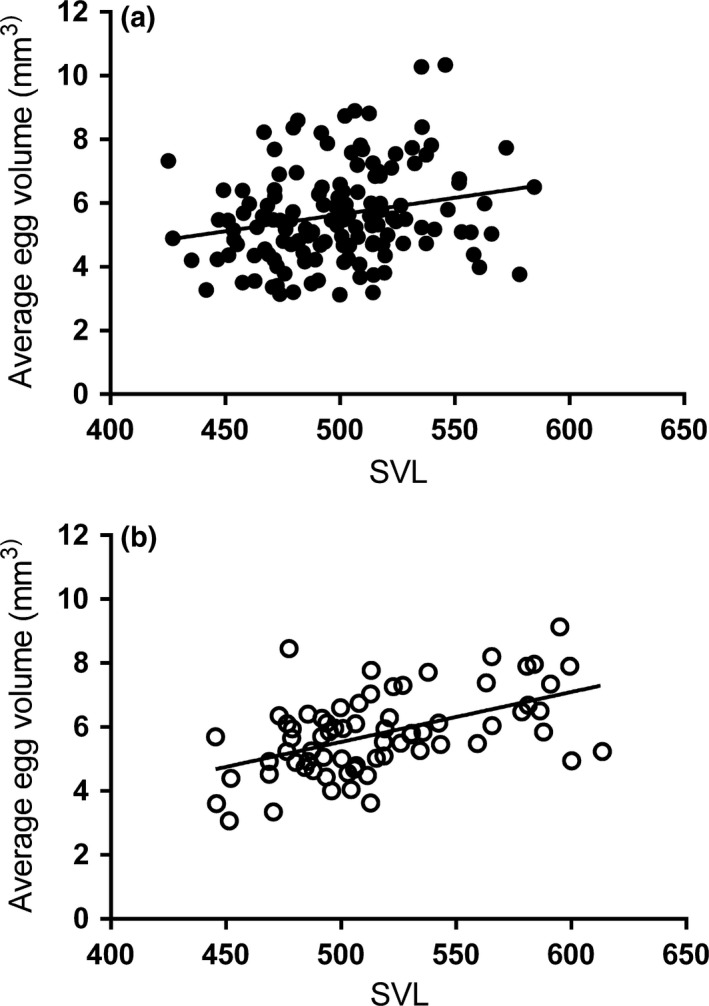
Relationship between average egg volume as estimated by ultrasonography and body size (SVL) in (a) *Thamnophis elegans* and (b) *Thamnophis sirtalis*, adjusted for other variables in the model described in Table 2

Egg volume also varied with source population, with comparisons of least‐square means showing PAP and NAM to have larger eggs ($\hat{x}\pm SE:6.40\pm 1.19$ and 6.60 ± 0.33 cm^3^) than MAH and SUM (5.60 ± 0.22 and 5.50 ± 0.34 cm^3^); PVM egg volume was intermediate (6.00 ± 0.38 cm^3^). Larger females had larger eggs, although the species x body size interaction revealed that this relationship differed between *T. sirtalis* and *T. elegans*, with *T. sirtalis* showing a steeper slope between mean egg volume and body size than *T. elegans* (*T. elegans*: slope = 0.010 ± 0.004; *T. sirtalis:* slope = 0.016 ± 0.003) (Figure 7). Females with larger litters tended to have smaller eggs. (a) *T. elegans* (slope = 0.010 ± 0.004) and (b) *T. sirtalis* (slope = 0.016 ± 0.003).

## 4 DISCUSSION

Our study indicates that in spite of sharing the same microhabitats and being dependent upon the same primary prey, two closely related species of snake exhibit marked differences in developmental life history (Table 3). First, we found that pregnant *T. sirtalis* females in this region had embryos at more advanced developmental stages than *T. elegans* females over the study period for all 3 years (Figure 2). Second*,* while both species showed a difference in mean egg volume between early and late developmental stages, the difference in volume was significantly less pronounced in *T. sirtalis* than in *T. elegans* (27% vs. 68% increase, respectively; Figure 3). Our primary method involved sampling a population cross‐section of females with embryos in different stages; however, repeated measures from a few female snakes showed a similar pattern, with *T. sirtalis* embryos showing only a modest change in volume over roughly a week's time in contrast to a more substantial change in *T. elegans* (2% in 8 days vs. 27% in 7 days, respectively). We interpret each of these findings in more detail below and explore their ramifications for future study of development in the wild.

**Table 3:** Comparison of ecology, life‐history, and developmental variables between *T. elegans* and *T. sirtalis* pregnant females in this study. For continuous variables, means and standard errors of the means are given. Egg volume means are based on eggs in early developmental stages

	*Thamnophis elegans* western terrestrial garter snake	*Thamnophis sirtalis* common garter snake
Habitat	Grassy meadow	Grassy meadow
Primary prey	Anurans, fish, leeches	Anurans, fish, leeches
Body size	504 ± 5 mm	538 ± 6 mm
Litter size	7.2 ± 0.2 eggs	10.8 ± 0.4 eggs
Egg volume	4.6 ± 0.2 m^3^	5.6 ± 0.6 m^3^
Developmental phenology	Delayed	Advanced
Maternal provisioning	Greater	Less

### 4.1 Developmental phenology

A potential proximate cause of species‐specific differences in developmental phenology could be variation in responsiveness to environmental cues for Spring emergence. Temperature is thought to be one of the major environmental cues stimulating emergence from hibernacula and commencement of spring reproductive activities in reptiles (Krohmer & Lutterschmidt, 2011). In our study system, *T. sirtalis* adults may emerge at cooler springtime temperatures, allowing eggs to be fertilized and ovulated at an earlier date than those of *T. elegans*. The fact that *T. sirtalis* has a more extensive northerly geographic range than any other reptile (Logier & Toner, 1961) may indicate a greater tolerance for cooler temperatures than *T. elegans*. Alternatively, if the two species do not differ with regard to responsiveness to seasonal cues, this would suggest that *T. sirtalis* embryos have the capacity to develop more rapidly in this region, whether due to relatively fixed genetic factors, or different gene by environment interactions than are present in *T. elegans*.

Whatever the cause, more advanced development in June for *T. sirtalis* during the 3 years of our study is likely linked to earlier parturition. Data from captive animals in 2012 showed that *T. sirtalis* from our study populations gave birth significantly earlier, on average, than *T. elegans* (August 6 ± 6 days vs. August 31 ± 15 days; K.C.Chism, A.M. Sparkman, and A.M. Bronikowski). Timing of gestation and hatching/birth can be a critical factor in determining juvenile survival (e.g., Uller & Olssen, 2010; Lorioux et al., 2013). There may be nuanced differences between the two species in neonate requirements for development and foraging time prior to winter hibernation, such that selection (past or present) has favored a developmental schedule that resulting in early parturition in *T. sirtalis*.

While all five meadows in this study exhibited similar habitat types—grassy shorelines and standing water with aquatic prey—population differences in developmental stage suggest that both species are influenced by environmental factors, such as temperature and precipitation, that vary among sites. For instance, meadows at low elevations (PAP: 1,645 m; PVM: 1,730 m) had only 49% of *T. elegans* and 12% of *T. sirtalis* females with embryos at early developmental stages, in comparison with 71% of *T. elegans* and 28% of *T. sirtalis* at higher elevations (SUM: 1,890 m; NAM: 1,915 m; MAH: 2065 m). This pattern was apparent in both species, and consistent with data from captive snakes, where low‐elevation PAP females gave birth earlier than high‐elevation MAH females (August 13 ± 15 days vs. August 24 ± 15 days, respectively) in 2012 (Chism, Sparkman & Bronikowski, unpubl. data). Populations at lower elevations experience earlier spring thaw, which may result in early emergence, earlier mating, and/or more rapid early embryonic development. Thus, while species differences in phenology are found throughout this region, regardless of microhabitat differences among populations, there is heterogeneity in intraspecific developmental phenology across populations that is worth further investigation.

Although recent climate change has been associated with changes in reproductive phenology in a variety of species, we found no significant difference in distribution of females with embryos at different developmental stages across years. This is in spite of the fact that 2015 and 2016 were much warmer years with earlier rise in Spring temperatures than in 2017 (e.g., mean April temperatures: 2015: 17°C; 2016: 19°C; 2017: 15°C; Station USC00048702, National Climate Data Center). That said, there were 13 *T. elegans* females with preovulatory vitellogenic follicles in 2017, a stage we did not observe in either of the previous years. This could suggest that some females are later to ovulate in years with cooler Spring temperatures. Alternatively, it may be that some females are able to begin vitellogenesis in 1 year, but will not have energetic reserves to complete it until the following year. In this case, observing some females with preovulatory vitellogenic follicles would not necessarily indicate a slight change in phenology so much as annual energetic constraints. Monitoring for additional years over a diversity of environmental conditions, and with finer categorization of developmental stages is needed to establish whether developmental phenology shifts over time within populations (as population differences would suggest), or is relatively canalized.

### 4.2 Maternal provisioning

We found strong evidence of maternal provisioning by both species of garter snake. We hypothesize that the change in mean egg volume across developmental stages we observed is primarily due to placental water uptake, as proposed in other viviparous reptiles (e.g., Dauphin‐Villemant & Xavier, 1986; Lourdais et al., 2015). Why *T. elegans* embryos should experience a greater transfer of water than *T. sirtalis* embryos is unclear. Interestingly, study of placentation in garter snakes has not uncovered notable differences in morphology between *T. sirtalis* and *T. ordinoides* or *T. radix* (the latter both closely related to *T. elegans*) that would predict such a difference (Blackburn & Lorenz, 2003a,b; Blackburn et al., 2002)—although the underlying morphological differences may be nuanced, and potentially even variable among populations. At some point in its evolution since the last common ancestor with *T. sirtalis*,* T. elegans* may have experienced environmental conditions that selected for greater maternal transfer of water during development, fine‐tuning maternal–fetal interactions in the already‐viviparous state common to all garter snakes. Alternatively, this feature may have been derived more recently in *T. elegans* populations residing within this particular geographic region.

We did find that while both species showed evidence of a similar trade‐off between egg size and number (i.e., where females with larger litters tended to have smaller eggs), there was a stronger positive relationship between body size and egg number in *T. sirtalis* than in *T. elegans* (Figure 7). This suggests that while there may be energetic constraints underlying the trade‐off between egg size and number, physical space is an additional constraint on egg size that is more pronounced in *T. sirtalis*. If selection (past or present) has favored a life‐history strategy in *T. sirtalis* that involves maximization of number of offspring in the body cavity, it may be that a trade‐off with offspring size is expressed via maternal water allocation. One study in the viviparous aspic vipers (*Vipera aspis*) reported that larger litters resulted in less embryonic sac fluid per offspring (Bonnet et al., 2017). *Thamnophis sirtalis* females did have significantly larger litters on average than *T. elegans* females in this sample, controlling for body size (Table 3). Furthermore, anecdotal data involving recapture of one *T. elegans* female after 21 days suggests that the increase in egg volume may result in increasingly more tightly packed eggs (Figure 6). If *T. sirtalis* eggs are already more tightly packed to begin with, this may disallow extensive water acquisition by embryos during gestation. Conversely, if *T. elegans* females are unable to maximally utilize their body cavity for reproduction—perhaps due to energetic constraints (note that different populations in this study had significantly different mean egg volumes, which may indicate energetic constraints)—the trade‐off between water allocation and offspring size may have been relaxed, allowing selection to favor more advanced placentotrophy (i.e., a greater degree of maternal provisioning via placenta). Future study of a greater number and diversity of garter snakes and other closely related viviparous clades would be useful in testing hypotheses regarding the interacting effects of phylogeny, body size, and litter size on changes in egg volume over development, to document the extent of variation within the viviparous reproductive mode, and the degree to which these relationships are canalized or plastic.

## 5 CONCLUSION

In this study, we describe a type of natural experimental system where two sympatric, closely related species experience many of the same environmental fluctuations, and yet differ in key aspects of developmental life history. Furthermore, we document intraspecific population differences in developmental phenology evident even on this small geographic scale. In general, this study provides a foundation for future work using ultrasonography in free‐living animals to document patterns of maternal provisioning in the evolution of viviparity, and potential impacts of fluctuations in water availability on maternal/fetal interactions. Our research also demonstrates the usefulness of ultrasonography for investigating the dynamics of developmental phenology in the wild, in a manner that may be suitable for a wide range species, providing valuable information for demographic and evolutionary analyses, and effects of environmental change that would otherwise be difficult to discern.

## CONFLICT OF INTEREST

None declared.

## AUTHOR CONTRIBUTIONS

AMS conceived and designed the study. All authors participated in the field work. AMS and KJC compiled and analyzed the data. AMS wrote the manuscript with contributions from DAWM; other authors provided editorial advice.

## DATA ACCESSIBILITY

Data are archived in Mendeley (Sparkman, 2017).
